# Prevalence of depression among students at a Sri Lankan University: A study using the Patient Health Questionnaire-9 (PHQ-9) during the COVID-19 pandemic

**DOI:** 10.1186/s12889-023-15427-y

**Published:** 2023-03-20

**Authors:** Ayanthi Wickramasinghe, Birgitta Essén, Rajendra Surenthirakumaran, Pia Axemo

**Affiliations:** 1 Department of Women and Children’s Health, International Maternal and Child Health unit (IMCH), Uppsala University, MTC-huset, Dag Hammarskjölds väg 14B, Uppsala, 752 37 Sweden; 2 Department of Community and Family Medicine, Faculty of Medicine, University of Jaffna, Jaffna, Sri Lanka

**Keywords:** Major depressive disorder, Mental health, PHQ-9, University students, South Asia, Public health, COVID-19

## Abstract

**Background:**

The prevalence of mental health disorders is known to be high among university students globally. Currently there are only a few studies on depression among university students in Sri Lanka. The aim of this study was to screen for the prevalence of Major Depressive Disorder (MDD) and other forms of depression, and to evaluate the factors associated with MDD.

**Methods:**

A cross sectional survey using the Patient Health Questionnaire (PHQ-9) was conducted among 637, second-year students from the faculties of Management Studies & Commerce, Science and Medicine at the University of Jaffna, during the Coronavirus (COVID-19) pandemic. Bivariate associations were assessed using chi-squared tests. Logistic regression was used to evaluate factors associated with any type of ragging.

**Results:**

MDD was considered to have been experienced by 31% of the students. From all three faculties, 70% of the students claimed to have experienced some form of depression ranging from mild to severe. The factor associated with MDD was the students’ ethnicity.

**Conclusion:**

Due to the high MDD risk among university students, it is imperative to develop psychosocial interventions to ensure early detection of mental health disorders and provide adequate support to safeguard this vulnerable population.

## Introduction

Depression is a common disorder that affects around 280 million people globally and is a leading cause of disability. By 2030, projections by the World Health Organization (WHO) foresee major depression to be the primary cause for non-fatal burden of disease worldwide [1]. The average age of onset of depression is around 24 years [1, 2]. Depression in young adults, including university students is one of the most common disorders observed in these groups and is a serious public health concern [3, 4]. According to a study by UNICEF carried out in 21 countries, in the first half of 2021, self-reported depression rates among the age group 15–24 years was approximately 19% [5]. A global systematic review of the prevalence of depression among university students found prevalence rates between 10 − 85%, with a weighted mean of 30.6% [6]. Depression among university students is known to cause several adverse consequences. It not only disrupts academic performance [7] and social development [8], but also increases university dropouts [9], alcohol and substance abuse [10] and increases the risk of suicide [9, 11]. University students are often in a critical phase of life, undergoing transitions both positive and negative. Most students are away from home for the first time, start living in hostels or student accommodation with other students, are experiencing romantic relationships, seeking independence with reduced adult supervision and taking care of themselves; these situations can often be overwhelming and could often lead to increased stress, anxiety and depression [8, 12].

In Sri Lanka, an increased prevalence of depression and other mental health problems have been recognized in this group. The few studies that have been conducted on depression and anxiety among university students have shown a high rate of depressive symptoms [12–15] with one study indicating emotional disorders to be as high as 63% among undergraduates [16]. The contributing factors mentioned were; difficulty gaining entrance to universities, increased expectations from parents to graduate from universities, being under pressure to perform well, maintaining family honor, as well as dealing with exam stress, financial difficulties, overcrowding in universities, insecurities related to employment opportunities, and difficulties to enter the desired job market [13, 16].

Another contributing factor could be an initiation ritual, extended in time termed as “ragging” that is carried out in Sri Lankan universities. New entrants undergo ragging, a practice similar to hazing, where they are often exposed to emotional/psychological, physical, and sexual abuse [16–21]. This practice which is rampant in local universities, has also been recognized as a cause of mental health disorders among students and has even led to suicides [13, 16, 22, 23]. Among several studies conducted by our research team, a quantitative study at the University of Jaffna, demonstrated a high prevalence of ragging (59%) and self-perceived mental health problems among students [24], and qualitative studies with the students [25] and individuals attached to the university also revealed the negative effects ragging had on students mental health.

Sri Lanka is multicultural, multilingual, and multiethnic, consisting of an ethno-religious blend of Sinhalese (75%), Tamils (11%), Moors/Muslims (9%), and other groups (5%) [26]. The ethnic-religious groups are regionally segregated with the Northern province predominated by the Tamil Hindus; the rest of the country is mainly Sinhalese Buddhists, while the Muslim population is spread throughout. Sri Lanka is still recovering from the destruction incurred by its 27-year civil war between the LTTE (Liberation Tigers of Tamil Elam) and the Sri Lankan government [27] that occurred in the North-East of the country.

Education worldwide was disrupted when the WHO declared coronavirus (COVID-19) caused by SARS-CoV-2, as a pandemic in March 2020, [28]. To control the spread of the disease many countries implemented measures such as social-distancing, closing public spaces, shutting down transport systems and imposing lockdowns. Sri Lanka suffered three major surges of COVID-19 cases that led to several lockdowns in the country [29]. In March 2020, a government decision was taken to close all education institutions, including universities. They were gradually re-opened from July 2020, on a limited scale, with priority to the final-year students. During 2021 there were several periodic closures of these institutes due to repeated COVID-19 waves in the country [28, 30]. During the pandemic, education was delivered via online platforms, as most universities were closed or only allowed to function at 50% of student capacity. Some students were allowed to attend practical and laboratory sessions, and examinations but had to remain within the student hostels for designated periods of time [28, 30].

The COVID-19 pandemic has impacted the mental health of students globally. Apart from the usual stress of exams and other university-related issues, students have had to deal with financial issues cause by the pandemic as well as uncertainties related to future employment. Several global studies among university students have shown an increase in mental health disorders such anxiety, depression, stress, and fear, due to the COVID-19 pandemic [30–33]. Similarly, students in Sri Lankan universities may have experienced elevated levels of depression and anxiety during the COVID-19 pandemic due to the numerous lockdowns for extended periods of time which could have led to uncertainties regarding their education and future. The known serious health consequences following a COVID-19 infection, could also be a stressor for the students own health and their families health. These factors could have made the university students in Sri Lanka more susceptible to mental health disorders apart from stressors such as pressure to perform and academic stress faced by university students in other countries. Studies on the mental health status among Sri Lankan University students during the COVID-19 pandemic are limited or nonexistent thus far.

Although a few studies on mental health disorders on students exist [13, 15, 34, 35], there is a lack of research among Sri Lankan universities located in the Northern province of Sri Lanka, especially concerning students of the three main ethnicities.

This study is also a part of a larger study on violence among students at the University of Jaffna. The findings from these studies indicated that more than fifty precents of the students claimed self-perceived health consequences such as anxiety and depression [24], and interviews with the students also revealed high levels of stress and anxiety [25]. This demonstrated the urgent need to carry out an exploratory study to determine the self-reported prevalence of depression among the students of the University of Jaffna as no baseline data was available to address this issue.

The aim of this study was to screen for self-reported prevalence of Major Depressive Disorder (MDD) and other forms of depression, and to evaluate factors associated with MDD among the students from the faculties of Management Studies & Commerce, Science, and Medicine, at the University of Jaffna, in a study conducted during the COVID-19 pandemic. Our prediction was that there could be a difference in MDD risk among students of different ethnicities. We assumed that the Tamil students were mostly from the Northern province, and traveled to university from home, were closer to their families and friends, were accustomed to the surroundings and life style which could result in lower rates of depression as compared to the students of other ethnicities who belonged to other parts of the country.

## Materials and Methods

### Study design and setting

An explorative study, using a cross-sectional survey, was conducted among 637 second-year students at the University of Jaffna, in March 2021.

The University of Jaffna is situated in the northern peninsula of Sri Lanka. Established in 1974, the University of Jaffna has 10 faculties, including Agriculture, Science, Arts, Business Studies, Engineering, Graduate Studies, Management Studies & Commerce, Medicine, Science and the faculty of Technology [36]. The University of Jaffna has almost similar numbers of students of each ethnic background; Sinhalese, Tamil, and Moors/Muslims, unlike other universities where the majority are Sinhalese. Hence, the University of Jaffna was chosen as the study site due to the student composition.

### Study population

All second years students from the faculties of Management Studies & Commerce, Science and Medicine were invited to participate in the study. These faculties were chosen due to their diverse mix of students from different ethnicities and these faculties being considered as large faculties. Second-year students were chosen to maintain uniformity among participants. Except for the Medical faculty the other two faculties consist of three-year programs. This was another consideration. The third-year students were excluded as the results could have been biased due to final year academic pressure and exams. The first-year students were also excluded as they may have been more recently been exposed to ragging, which could have affected their mental health.

### Survey instrument and data collection

The survey instrument used was a self-administered Patient Health Questionnaire with 9 items (PHQ-9) to screen for students self-reported signs of depression. The PHQ-9 is a widely used simple, effective, and reliable tool for screening and evaluation of the severity of depression [37, 38]. It has been validated and used for screening purposes in different settings in Sri Lanka [39]. The PHQ-9 has been translated into Sinhalese and Tamil languages for the use in the National Mental Health Survey of Sri Lanka. The questionnaires contained PHQ-9 questions in English, Sinhalese and Tamil and included questions on the students’ background characteristics.

Research assistants distributed the questionnaires to students from the faculties of Management Studies & Commerce and Science following a routine test, and the Medical faculty students received it after a compulsory lecture. The aim of the study was explained to all students and a verbal consent was obtained. Students were informed that participation was completely voluntary and they could answer the questions in any of the three languages they preferred. Students were also informed that they could withdraw without consequence at any time during the study. The students participating in the survey were offered information on where to seek support services, counselling and medical services at the university.

The initial research plan was to distribute the questionnaires to students in early 2020, before the world became aware of the COVID-19 pandemic. Due to COVID-19 being declared a pandemic and due to the university lockdowns, the distribution of questionnaires were delayed until December 2020.

### Outcome measures

The PHQ-9 consists of nine Likert scale questions [38]. Mental health status was measured on depression symptoms over the last 2 weeks, with responses of; 0 = ‘not at all’, 1 = ‘several days’, 2 = ‘more than half the days’, and 3 = ‘nearly every day’. The item scores are summed-up to produce a total score between 0 and 27. The PHQ-9 total score is divided into the following categories of increasing severity of depressive symptoms: 0–4 = ‘none’, 5–9 = ‘mild’, 10–14 = ‘moderate’, 15–19 = ‘moderately-severe’, and 20–27 = ‘severe’. Students who scored 10 or above, were considered to have reported symptoms of moderate, moderately-severe or severe depression and categorized as having experienced symptoms of Major Depressive Disorder (MDD).

### Covariates

Questions on students’ background characteristics were as follows: Sex categorized as male or female, ethnicity classified as Sinhalese, Tamil, and Moor/Muslim, education level of father’s/mother was categorized to below or above 11 years of schooling (compulsory years of schooling).

### Analysis

From the total number of second-year students enrolled (N = 687) in the three faculties, 674 students responded to the questionnaires. Despite having a response rate of 98%, only 637 students were included in the study due to incomplete questionnaires by 37 students. A questionnaire was considered complete if the participant had responded to at least seven out of nine questions.

Descriptive characteristics of the students as well as levels of severity of depression are presented as frequencies and percentages. Chi-squared tests were used to compare proportions and to evaluate bivariate association between characteristics of the students, faculty and MDD. Logistic regression models were used to evaluate factors associated with MDD among the students. The results are presented in two models. The first unadjusted model was followed by a second adjusted model including all factors considered significant in univariate analysis with *p* < 0.20 [40]. Odds ratios (OR) with 95% confidence intervals were calculated. Statistical significance was considered if *p <* 0.05. The data was analyzed using the statistical software, “RStudio” (3.5.2 Eggshell Igloo).

## Results

The percentage of female students (59%) was higher than male students. The age span was between 21 and 30 years, mean 23 years (Standard deviation 0.98). There were almost equal numbers of Sinhalese and Tamil students. Sinhalese students were significantly (*p = 0.02*) more likely to experience MDD. Most of the students had parents with more than 11 years of schooling. A majority of the students belonged to the faculty of Management Studies & Commerce although this was not significant (*p = 0.25*). (Table 1).

**Table 1:** Descriptive characteristics of the students from the faculties of Management Studies & Commerce, Science and Medicine

Variable	Number of students (N = 637)	Students without MDD (N = 446)	Students with MDD* (N = 191)	P value**
**Sex**				
Female	378 (59%)	271 (72%)	107 (28%)	0.30
Male	259 (41%)	175 (68%)	84 (32%)	
**Age**				
< 22 years	124 (19%)	93 (75%)	31 (25%)	0.09
23 years	226 (36%)	166 (73%)	60 (27%)	
24 years	178 (28%)	113 (63%)	65 (37%)	
> 25 years	109 (17%)	74 (68%)	35 (32%)	
**Ethnicity**				
Moor/Muslim	46 (7%)	39 (85%)	7 (15%)	**0.02**
Sinhalese	290 (46%)	191 (66%)	99 (34%)	
Tamil	301 (47%)	216 (72%)	85 (28%)	
**Father’s** **schooling**				
< 11 years	231 (36%)	153 (66%)	78 (34%)	0.14
> 11 years	406 (64%)	293 (72%)	113 (28%)	
**Mother’s schooling**				
< 11 years	199 (31%)	130 (65%)	69 (35%)	0.09
> 11 years	438 (69%)	316 (72%)	122 (28%)	
**Faculty**				
Management^#^	305 (48%)	204 (67%)	101(33%)	0.25
Science	202 (32%)	148 (73%)	54 (27%)	
Medicine	130 (20%)	94 (72%)	36 (28%)	

*MDD - Major Depressive Disorder (PHQ-9 score ≥ 10). **Chi-squared was used to compare proportions. Values that are significant at the p < 0.05 level are shown in bold. #Management Studies & Commerce faculty.

### Forms of depression

In the three faculties, 31% of the students were considered to have experienced MDD. Of all students, the students from the faculty of Management Studies & Commerce claimed to have experienced most self-reported signs of MDD (15%). Symptoms of mild depression were reported by 39% of the students. Therefore 70% of the students from across these faculties claimed to have experienced different forms of depression ranging from mild to severe, while 30% of the students did not have any form of depressive symptoms.

Among all the students, a highest MDD risk was seen among females (17%) as compared to males (13%) although there was no significant *(P = 0.22)* difference. Looking at the different faculties, females claimed to have experienced more MDD in the Management Studies & Commerce (11%) and Medical (3%) faculties, while a higher percentage of males claiming signs of MDD were seen in the Science faculty (5%).

### Factors associated with major depressive disorder

In the unadjusted analysis, ethnicity was significantly associated with experiencing symptoms of MDD among the students (Table 2). Students who belonged to the Sinhalese ethnicity were at higher odds of experiencing MDD compared to the students who were ethnically Moor/Muslim (UOR 2.90, 95% of CI: 1.33–7.30). In the adjusted model, ethnicity continued to be associated with MDD, where Sinhalese students sustained increased odds of experiencing MDD compared to Moor/Muslim students (AOR 2.87, 95% of CI: 1.30–7.25).

**Table 2:** Factors associated with Major Depressive disorder (MDD) among the students from the faculties of Management Studies & Commerce, Science and Medicine

Variable	UOR	P value (95% CI)	AOR	P value (95% CI)
**Sex**				
Female Male	Reference 1.22	Reference 0.16 (0.86–1.71)	Reference 1.23	Reference 0.41 (0.80–1.59)
**Ethnicity**				
Moor/Muslim Sinhalese Tamil	Reference 2.90 2.19	Reference 0.01 (1.33–7.30) 0.06 (0.10–5.53)	Reference 2.87 2.11	Reference 0.01 (1.25–6.93) 0.07 (0.97–5.44)
**Father’s schooling**				
<11 years >11 years	Reference 0.76	Reference 0.06 (0.53–1.07)	Reference 0.91	Reference 0.58 (0.57–1.47)
**Mother’s schooling**				
<11 years >11 years	Reference 0.73	Reference 0.06 (0.51–1.04)	Reference 0.76	Reference 0.24 (0.47–1.23)
**Faculty**				
Management^#^ Science Medical	Reference 0.74 0.77	Reference 0.42 (0.50–1.09) 0.09 (0.49–1.21)	Reference - -	Reference - -

UOR-unadjusted odds rations, AOR-adjusted odds ratio; CI-confidence interval; AOR (CI) values significant at the p < 0.05 level are shown in bold.

^#^Management Studies & Commerce faculty.

## Discussion

This study is among one of the first studies on depression screening using a PHQ-9 questionnaire, among university students of all ethnicities in a university in the Northern province of Sri Lanka, during the COVID-19 pandemic. The results indicated that almost one-third of the second-year students reported experiencing MDD in the faculties of Management Studies & Commerce, Science and Medicine, at the University of Jaffna. Around 70% of the students reported symptoms of depression ranging from mild to severe. The main factor associated with MDD was the Sinhalese ethnicity.

Our study found a high risk for MDD (31%) among the students in these three faculties. A study among students from the University of Colombo using a PHQ-9 questionnaire found a prevalence rate of 9.3% [13], which was lower than our study. Higher prevalence rates were seen in a study among the first-year students at the university of Ruhuna, where ‘elevated depressive’ symptoms were reported among 76% of medical students and 60% of non-medical students [14]. In another study, among medical students at the same university, severe psychological distress was seen among 40.4% of the students [34]. Depression rates among nursing students in the university of Peradeniya were also found to be as high as 51% [35]. The variation in the prevalence rates in previous studies conducted in Sri Lanka could be due to the differences in questionnaires used, different analytical methods, different locations of the universities, student compositions, and different academic years. Furthermore, all these studies were conducted prior to the COVID-19 pandemic, which may have contributed to the higher risk of MDD seen among the participants of our study due to the uncertainties caused by this new disease and the impact it may have on their education and future.

International research, prior to the COVID-19 pandemic, that used the PHQ-9 questionnaire also demonstrated varied prevalence rates of depression. A study in England showed, severe depression rates among medical and non-medical students were found to be 5.6% and 12.7% respectively, while moderate depression was 10.8% in medical and 17.7% in non-medical students, which were lower than our findings [41]. In contrast higher prevalence rates than in our findings were seen in Brazil where 32% of first year students, from all the faculties presented with symptoms of MDD [42]. In Bangladesh, prevalence rates of moderate to severe depression were 47% among students independent of academic year in two public universities [43]. The higher prevalence of MDD symptoms in our study, could be owed to the stress factors caused by the COVID-19 pandemic.

No gender difference was found in our study. This result is comparable to a few Sri Lankan studies showing no gender difference [13, 44], indicating that both male and female university students are at equal risk of depression. In contrast, several other studies that had found a gender difference, showed an elevated prevalence of depression among women [6, 8, 14, 34, 42, 43]. The increased risk of depression among females in low- and middle-income countries could be attributed to gender and social inequalities women face throughout their life course [43]. The lack of gender differences in our study could have several reasons; this study was carried-out during COVID-19 pandemic where both males and females were worried about their health and future as this was a new and unknown disease [30]. Also, both young men and women are trying to adapt to the changes university life brings and face difficulties being away from home [12]. According to literature ragging is known to be more severe in males, as a consequence, males may have a high MDD risk [24, 25, 45]. Males, in contrast to females, in Sri Lankan universities have been known to use more alcohol and substances which could also lead to a higher number of males experiencing symptoms of MDD [45]. These reasons may have led to there being no difference in the reported prevalence of MDD between the sexes.

The prevalence of MDD reported among the students in our study was not affected by belonging to a certain faculty. Most research conducted among university students has focused on Medical students as they are believed to experience more mental health disorders than their counterparts due to the stressful and demanding nature of the medical curriculum [14, 15, 34, 46, 47]. In Sri Lanka, medical students have a guaranteed job in the public sector upon completion of their education, with a higher earning capacity than students in humanities, management and science [44]. The job insecurity and the uncertainty of their future faced by students of other faculties, especially during the COVID-19 pandemic [28] may have been an equalizing factor for similar rates of depression among all the faculties in our study. Another reason there was no significant difference between the faculties could be due to the situation when the data was collected. Data collection in the Faculties of Management studies & Commerce and Science was carried out following a routine test where the students could have experienced more stress and anxiety, as opposed to the data collection in the Faculty of Medicine which occurred after a compulsory lecture. This factor could have led to the false leveling of the prevalence rates among the students of these three faculties.

Studies conducted in Sri Lanka [34, 35], Bangladesh [43], and Malaysia [48] indicated that the prevalence of depression increased with the students’ academic year. This could be due to increased work load and stress associated with the progression of studies [43]. Contrary to this, a Sri Lankan study [13], demonstrated that students in lower academic years suffered more MDD compared to 4th year students, which was similar to findings in a global systematic review [6]. Another study also demonstrated higher depression rates among first-year students in Sri Lanka [14]. First-year students are most often more exposed to ragging during their first year in university. [21]. Ragging is known to affect students both physically and psychologically and takes a toll on their mental health [13, 23]. Apart from ragging, first-year students are away from their families, must adapt to their new life in the university and cope with the stress of course work and exams, which could contribute to an increase in mental health disorders in this population.

Our study demonstrated a significant association between MDD and ethnicity, indicating that the Sinhala students were more likely to be affected by MDD than the Moor/Muslim students. Previous studies on mental health among university students or the general population in Sri Lanka have not estimated the prevalence of depression in each ethnicity, therefore baseline values are not available for comparison. Jaffna, a predominantly Tamil area, has several cultural differences; their customs, style of dressing, and food, and most Sinhalese students have a challenging time adapting to this environment [25]. Most of the Tamil, and some Moor/Muslims’ students live outside the campus with families or relatives while Sinhalese students who are not native to this area are unable to travel home during most weekends because of economic cost and the distances. Being unable to visit their families and adapting to a new area as well as university life may be another reason Sinhalese students report more MDD compared to Moor/Muslim students and Tamil students in the University of Jaffna. According to Kuruppuarachchi [44], students having to adapt to unfamiliar environments that are vastly different from what they are familiar with, suffer more psychological distress. Similarly, studies also show that students living in hostels or student housing are more likely to experience depression than students living with their families [13, 14]. Sinhalese students who remain in hostels during the weekends with less staff supervision, are more susceptible to ragging from the senior Sinhalese students who also remain there [25]. In comparison, ragging also occurs at a lesser frequency amongst the Moor/Muslim students [24, 25]. This could be one reason Sinhalese students are more vulnerable to MDD.

The data collection of this study occurred during the COVID-19 pandemic after a prolonged lockdown. While most lectures were held online, students at the University of Jaffna were allowed to attend exams and participate in practical training in the hospital. Each faculty was given a certain time-frame when students could attend the exams on site. Students were extensively tested for COVID-19 and had to abide by strict rules to prevent the spread of the infection. During this period, students had to remain in the hostel and were not allowed to go outside or visit family. Students who contracted COVID-19 were isolated and treated at the Jaffna General Hospital.

The strict regulations students had to adhere to, the fear of falling ill and the uncertainty for their future, triggered by the COVID-19 pandemic, may have contributed to the high prevalence rates of MDD seen in our study. International studies conducted during the COVID-19 pandemic demonstrated an increase in the prevalence of mental health disorders among the general population [49] and university students [32, 50, 51].

### Strengths and limitations

A limitation of this study was that the cross-sectional study design does not allow the determination of a causal relationship. Longitudinal studies are required to assess the relationship between different associated factors and MDD. Another limitation of the study was that we lacked knowledge of students’ history of depression or family history of mental illness before entering the university or if they were receiving treatment for any mental health disorders. Social desirability effect and subjectivity of answers could also have been a possible limitation as the students may have refrained from being completely truthful about a stigmatized topic as mental illness. Distributing the questionnaires to students from the faculties of Management Studies & Commerce and Science following a routine test, could have led to higher rates of MDD among these students.

A strength of our study was that this is one of the few studies that investigate the self-reported prevalence of depression among university students in Sri Lanka, an often-underrepresented group but vital for public health research. The study also tests the role of ethnic differences among university students, that to our knowledge has not been previously investigated. University of Jaffna the study site, has almost similar numbers of Sinhalese and Tamil students and Moor/Muslim students representing the main ethnicities which is uncommon for other Sri Lankan universities. Another strength of this study was the high response rate. This study is one of the few studies that was conducted during the COVID-19 pandemic and gave us further insight into the potential increase in mental health problems university students might face due to the disruption of normal life, health concerns, concerns regarding their education and future, and economic concerns, which could lead to elevated stress and anxiety in this group.

## Conclusion

This study demonstrates a high self-reported PHQ-9 scores (≥ 10) representing a high MDD risk among students of the University of Jaffna. Our findings also help gain a better understanding of the factors associated with depression and indicate the importance of screening this vulnerable population to intervene and counter the effects of depression and other mental health issues. Planning effective interventions and policies is essential to safeguard all university students. Early recognition of depression and other mental health disorders, ensuring appropriate counseling services for students and expanding existing counseling programs are vital. Creating a safe environment free of harmful ragging, initiation of stress management programs, mentorship programs, recreational facilities, proper resources and information on where to seek psychological help is also crucial towards maintaining students’ mental wellbeing.

## Data Availability

The data presented in this study are available on request from the corresponding author. The data are not publicly available due to sensitive nature of the study.

## References

[1] Malhi GS, Mann JJ, Depression.Lancet Lond Engl. 2018 Nov24;392(10161):2299–312. 10.1016/S0140-6736(18)31948-2 30396512

[2] Nihalani N, Simionescu M, Dunlop BW, Depression. Phenomenology, Epidemiology, and Pathophysiology. Depression. 2nd ed.CRC Press; 2010.

[3] Auerbach RP, Alonso J, Axinn WG, Cuijpers P, Ebert DD, Green JG Mental disorders among college students in the World Health Organization World Mental Health Surveys. Psychol Med [Internet]. 2016 Oct [cited 2022 Jun 11];46(14):2955–70. Available from: https://www.cambridge.org/core/journals/psychological-medicine/article/mental-disorders-among-college-students-in-the-world-health-organization-world-mental-health-surveys/10.1017/S0033291716001665 PMC5129654 27484622

[4] Ebert DD, Buntrock C, Mortier P, Auerbach R, Weisel KK, Kessler RC et al. Prediction of major depressive disorder onset in college students. Depress Anxiety [Internet]. 2019 [cited 2022 Jun 11];36(4):294–304. Available from: https://onlinelibrary.wiley.com/doi/abs/10.1002/da.22867 10.1002/da.22867 PMC6519292 30521136

[5] The State of the World’s. Children 2021 [Internet]. [cited 2022 Jun 11]. Available from: https://www.unicef.org/reports/state-worlds-children-2021

[6] Ibrahim AK, Kelly SJ, Adams CE, Glazebrook C. A systematic review of studies of depression prevalence in university students. J Psychiatr Res [Internet]. 2013 Mar 1 [cited 2022 Jun 11];47(3):391–400. Available from: https://www.sciencedirect.com/science/article/pii/S0022395612003573 10.1016/j.jpsychires.2012.11.015 23260171

[7] Hysenbegasi A, Hass SL, Rowland CR. The Impact of Depression on the Academic Productivity of University Students.J Ment Health Policy Econ. 2005;8. 16278502

[8] Adewuya AO, Ola BA, Aloba OO, Mapayi BM, Oginni OO. Depression amongst Nigerian university students. Soc Psychiatry Psychiatr Epidemiol [Internet]. 2006 Aug 1 [cited 2022 Jun 11];41(8):674–8. Available from: http://link.springer.com/article/10.1007/s00127-006-0068-9 10.1007/s00127-006-0068-9 16680408

[9] Arria AM, O’Grady KE, Caldeira KM, Vincent KB, Wilcox HC, Wish ED. Suicide Ideation Among College Students: A Multivariate Analysis. Arch Suicide Res [Internet]. 2009 Jul 16 [cited 2022 Jun 11];13(3):230–46. Available from: https://www.tandfonline.com/doi/abs/10.1080/13811110903044351 10.1080/13811110903044351 PMC2709750 19590997

[10] Serras A, Saules KK, Cranford JA, Eisenberg D (2010). Self-injury, substance use, and associated risk factors in a multi-campus probability sample of college students. Psychol Addict Behav.

[11] Eisenberg D, Hunt J, Speer N. Mental Health in American Colleges and Universities: Variation Across Student Subgroups and Across Campuses. J Nerv Ment Dis [Internet]. 2013 Jan [cited 2022 Jun 11];201(1):60–7. Available from: https://journals.lww.com/jonmd/Abstract/2013/01000/Mental_Health_in_American_Colleges_and.12.aspx 10.1097/NMD.0b013e31827ab077 23274298

[12] Read JP, Wood MD, Davidoff OJ, McLacken J, Campbell JF. Making the transition from high school to college: The role of alcohol-related social influence factors in students’ drinking. Subst Abuse [Internet]. 2002 Mar 1 [cited 2022 Jun 11];23(1):53–65. Available from: 10.1080/08897070209511474 10.1080/08897070209511474 12444360

[13] Amarasuriya SD, Jorm AF, Reavley NJ. Prevalence of depression and its correlates among undergraduates in Sri Lanka. Asian J Psychiatry [Internet]. 2015 Jun [cited 2021 Jan 1];15:32–7. Available from: https://linkinghub.elsevier.com/retrieve/pii/S1876201815000738 10.1016/j.ajp.2015.04.012 25998095

[14] Perera B (2011). Are medical undergraduates more vulnerable than their non-medical peers to develop depressive symptoms. Galle Med J.

[15] Dahanayake D, Rajapakse H, Wickramasinghe A, Chandradasa M, Rohanachandra Y, Perera S et al. Psychological wellbeing and mental health amongst medical undergraduates: A descriptive study assessing more than 1,000 medical students in Sri Lanka. Int J Soc Psychiatry [Internet]. 2021 Jun 18 [cited 2022 Jun 11];00207640211027211. Available from: 10.1177/00207640211027211 10.1177/00207640211027211 34144652

[16] Kathriarachchi ST, Ariyaratne CV, Jiffry MTM. Frequency of Emotional Disorders among University Entrants of a University in Sri Lanka. 2001 [cited 2022 Jun 11]; Available from: http://dr.lib.sjp.ac.lk/handle/123456789/1000

[17] Garg R, Ragging. A public health problem in India. Indian J Med Sci [Internet]. 2009 [cited 2021 Jan 14];63(6):263. Available from: http://www.indianjmedsci.org/text.asp?2009/63/6/263/53401 19602763

[18] Shakya DR, Maskey R. “Ragging”: What the medical students of a health institute from Eastern Nepal say? Sunsari Tech Coll J [Internet]. 2012 [cited 2020 Dec 9];1(1):27–32. Available from: https://www.nepjol.info/index.php/STCJ/article/view/8658

[19] Wajahat A. Harassment Due to Ragging. Procedia Soc Behav Sci [Internet]. 2014 Feb 1;113:129–33. Available from: https://www-sciencedirect-com.ezproxy.its.uu.se/science/article/pii/S1877042814000202

[20] Mamun MA, Hossain MdS, Griffiths MD. Mental Health Problems and Associated Predictors Among Bangladeshi Students. Int J Ment Health Addict [Internet]. 2022 Apr [cited 2022 Jun 15];20(2):657–71. Available from: https://link.springer.com/10.1007/s11469-019-00144-8

[21] Gunatilaka H, Ragging. Its Evolution and Effects: A Literature Review with a Special Reference to Sri Lanka. Int J Res Innov Soc Sci IJRISS [Internet]. 2019 Oct 1;2454–6186. Available from: http://dr.lib.sjp.ac.lk/handle/123456789/11918

[22] McCaslin SE, de Zoysa P, Butler LD, Hart S, Marmar CR, Metzler TJ et al. The relationship of posttraumatic growth to peritraumatic reactions and posttraumatic stress symptoms among Sri Lankan university students. J Trauma Stress [Internet]. 2009 Aug 1 [cited 2022 May 12];22(4):334–9. Available from: https://onlinelibrary.wiley.com/doi/10.1002/jts.20426 10.1002/jts.20426 19588514

[23] Premadasa IG, Wanigasooriya NC, Thalib L, Ellepola ANB (2011). Harassment of newly admitted undergraduates by senior students in a Faculty of Dentistry in Sri Lanka. Med Teach.

[24] Wickramasinghe A, Essén B, Ziaei S, Surenthirakumaran R, Axemo P. Ragging, a Form of University Violence in Sri Lanka—Prevalence, Self-Perceived Health Consequences, Help-Seeking Behavior and Associated Factors. Int J Environ Res Public Health [Internet]. 2022 Jan [cited 2022 Jul 28];19(14):8383. Available from: https://www.mdpi.com/1660-4601/19/14/8383 10.3390/ijerph19148383 PMC9318855 35886237

[25] Wickramasinghe A, Axemo P, Essén B, Trenholm J. Ragging as an expression of power in a deeply divided society; a qualitative study on students perceptions on the phenomenon of ragging at a Sri Lankan university. PLOS ONE [Internet]. 2022 Jul 11 [cited 2022 Jul 29];17(7):e0271087. Available from: https://journals.plos.org/plosone/article?id=10.1371/journal.pone.0271087 10.1371/journal.pone.0271087 PMC9273066 35816476

[26] Department of Census and Statistics. Census of Population and Housing 2011 [Internet]. 2012 [cited 2021 Mar 12]. Available from: http://www.statistics.gov.lk/PopHouSat/CPH2011/index.php?fileName=Activities/TentativelistofPublications

[27] Ganguly S. Ending the Sri Lankan Civil War. Daedalus [Internet]. 2018 Jan 1 [cited 2021 Nov 22];147(1):78–89. Available from: 10.1162/DAED_a_00475

[28] Hayashi R, Garcia M, Kaushalya T, Amaratunge S, Hewagamage KP. Sri Lanka-Progress and Remaining Challenges in Online Higher Education during the COVID-19 Pandemic [Internet]. 0 ed. Manila, Philippines: Asian Development Bank; 2022 May [cited 2022 Jun 13]. (ADB Briefs). Available from: https://www.adb.org/publications/sri-challenges-online-higher-education-covid-19

[29] COVID-19 Epidemiology. Sri Lanka [Internet]. [cited 2022 Jun 13]. Available from: http://epid.gov.lk/web/images/pdf/corona_monthly_summery/esummery-september.pdf

[30] Ilangarathna GA, Ranasinghe Y, Weligampola H, Attygalla E, Ekanayake J, Yatigammana S et al. A Comprehensive Overview of Education during Three COVID-19 Pandemic Periods: Impact on Engineering Students in Sri Lanka. Educ Sci [Internet]. 2022 Mar 11 [cited 2022 Jun 13];12(3):197. Available from: https://www.mdpi.com/2227-7102/12/3/197

[31] Islam S, Akter R, Sikder T, Griffiths MD. Prevalence and Factors Associated with Depression and Anxiety Among First-Year University Students in Bangladesh: A Cross-Sectional Study. Int J Ment Health Addict [Internet]. 2020 Mar 2 [cited 2022 May 10];1–14. Available from: https://link.springer.com/article/10.1007/s11469-020-00242-y

[32] Gritsenko V, Skugarevsky O, Konstantinov V, Khamenka N, Marinova T, Reznik A et al. COVID 19 Fear, Stress, Anxiety, and Substance Use Among Russian and Belarusian University Students. Int J Ment Health Addict [Internet]. 2021 Dec 1 [cited 2022 Jun 13];19(6):2362–8. Available from: 10.1007/s11469-020-00330-z 10.1007/s11469-020-00330-z PMC7241583 32837418

[33] Savitsky B, Findling Y, Ereli A, Hendel T. Anxiety and coping strategies among nursing students during the covid-19 pandemic. Nurse Educ Pract [Internet]. 2020 Jul 1 [cited 2022 Jun 13];46:102809. Available from: https://www.sciencedirect.com/science/article/pii/S1471595320303371 10.1016/j.nepr.2020.102809 PMC7264940 32679465

[34] Wimberly CE, Rajapakse H, Park LP, Price A, Proeschold-Bell RJ, Østbye T. Mental well-being in Sri Lankan medical students: a cross-sectional study. Psychol Health Med [Internet]. 2020 Dec 27 [cited 2022 May 12];0(0):1–14. Available from: 10.1080/13548506.2020.1858488 10.1080/13548506.2020.1858488 33356528

[35] Rathnayake S, Ekanayaka J, Depression. Anxiety and Stress among Undergraduate Nursing Students in a Public University in Sri Lanka. Int J Caring Sci [Internet]. 2016;9:1020–32. Available from: https://internationaljournalofcaringsciences.org/docs/31_rathnayaky_originial_9_3.pdf

[36] University of Jaffna [Internet]. University of Jaffna. 2020 [cited 2020 Dec 9]. Available from: http://www.jfn.ac.lk/

[37] Sun Y, Fu Z, Bo Q, Mao Z, Ma X, Wang C. The reliability and validity of PHQ-9 in patients with major depressive disorder in psychiatric hospital. BMC Psychiatry [Internet]. 2020 [cited 2022 May 17];20. Available from: https://www.ncbi.nlm.nih.gov/pmc/articles/PMC7525967/10.1186/s12888-020-02885-6 PMC7525967 32993604

[38] Kroenke K, Spitzer RL, The. PHQ-9: A New Depression Diagnostic and Severity Measure. Psychiatr Ann [Internet]. 2002 Sep [cited 2022 May 17];32(9):509–15. Available from: https://journals.healio.com/doi/10.3928/0048-5713-20020901-06

[39] Institute for Research & Development. National Mental Health Survey, Sri Lanka [Internet]. 2007 Mar [cited 2022 May 17]. Available from: https://www.ird.lk/mental-health-survey/

[40] Harrell F. Regression modeling strategies with applications to linear models, logistic regression, and survival analysis [Internet]. New York: Springer; 2001 [cited 2022 Jun 21]. Available from: https://link.springer.com/book/10.1007/978-3-319-19425-7

[41] Honney K, Buszewicz M, Coppola W, Griffin M. Comparison of levels of depression in medical and non-medical students. Clin Teach [Internet]. 2010 [cited 2022 Jun 23];7(3):180–4. Available from: https://onlinelibrary.wiley.com/doi/abs/10.1111/j.1743-498X.2010.00384.x 10.1111/j.1743-498X.2010.00384.x 21134179

[42] Flesch BD, Houvèssou GM, Munhoz TN, Fassa AG. Major depressive episode among university students in Southern Brazil. Rev Saúde Pública [Internet]. 2020 Jan 31 [cited 2022 Jun 11];54. Available from: http://www.scielo.br/j/rsp/a/abstract/lang=en 10.11606/s1518-8787.2020054001540 PMC6986868 32022140

[43] Koly KN, Sultana S, Iqbal A, Dunn JA, Ryan G, Chowdhury AB. Prevalence of depression and its correlates among public university students in Bangladesh. J Affect Disord [Internet]. 2021 Mar 1 [cited 2022 Jun 23];282:689–94. Available from: https://www.sciencedirect.com/science/article/pii/S0165032720332274 10.1016/j.jad.2020.12.137 33445093

[44] Kuruppuarachchi KA, Kuruppuarachchi KA, Wijerathne S, Williams SS (2002). Psychological distress among students from five universities in Sri Lanka. Ceylon Med J.

[45] Prevalence of Ragging and Sexual and Gender Based Violence [Internet]. 2022 [cited 2022 Apr 15]. Available from: https://www.unicef.org/srilanka/reports/prevalence-ragging-and-sexual-and-gender-based-violence

[46] Shakya DR, Dr, Shyangwa P, Shakya R, Agrawal C. Mental and Behavioral Problems in Medical Students of a Health Institute in Eastern Nepal.Asian J Psychiatry. 2011 Jul 1;4.

[47] Sultana N. Stress and Depression among undergraduate Medical Students of Bangladesh. Bangladesh J Med Educ [Internet]. 2011 [cited 2022 Jun 24];2(1):6–9. Available from: https://www.banglajol.info/index.php/BJME/article/view/18130

[48] Shamsuddin K, Fadzil F, Ismail WSW, Shah SA, Omar K, Muhammad NA et al. Correlates of depression, anxiety and stress among Malaysian university students. Asian J Psychiatry [Internet]. 2013 Aug 1 [cited 2022 Jun 24];6(4):318–23. Available from: https://www.sciencedirect.com/science/article/pii/S1876201813000592 10.1016/j.ajp.2013.01.014 23810140

[49] Santomauro DF, Herrera AMM, Shadid J, Zheng P, Ashbaugh C, Pigott DM et al. Global prevalence and burden of depressive and anxiety disorders in 204 countries and territories in 2020 due to the COVID-19 pandemic. The Lancet [Internet]. 2021 Nov 6 [cited 2022 Jun 11];398(10312):1700–12. Available from: https://www.thelancet.com/journals/lancet/article/PIIS01406736(21)02143-7/abstract 10.1016/S0140-6736(21)02143-7 PMC8500697 34634250

[50] Islam MA, Barna SD, Raihan H, Khan MNA, Hossain MT. Depression and anxiety among university students during the COVID-19 pandemic in Bangladesh: A web-based cross-sectional survey. PLOS ONE [Internet]. 2020 Aug 26 [cited 2022 May 10];15(8):e0238162. Available from: https://journals.plos.org/plosone/article?id=10.1371/journal.pone.0238162 10.1371/journal.pone.0238162 PMC7449469 32845928

[51] Najjuka SM, Checkwech G, Olum R, Ashaba S, Kaggwa MM. Depression, anxiety, and stress among Ugandan university students during the COVID-19 lockdown: an online survey. Afr Health Sci [Internet]. 2021 [cited 2023 Jan 31];21(4):1533–43. Available from: https://www.ajol.info/index.php/ahs/article/view/218774 10.4314/ahs.v21i4.6 PMC8889827 35283951

